# DNA methylation age of blood predicts future onset of lung cancer in the women's health initiative

**DOI:** 10.18632/aging.100809

**Published:** 2015-09-24

**Authors:** Morgan E. Levine, H. Dean Hosgood, Brian Chen, Devin Absher, Themistocles Assimes, Steve Horvath

**Affiliations:** ^1^ Human Genetics, David Geffen School of Medicine, University of California LA, Los Angeles, CA 90095, USA; ^2^ Center for Neurobehavioral Genetics, University of California Los Angeles, Los Angeles, California 90095, USA; ^3^ Department of Epidemiology and Population Health, Albert Einstein College of Medicine, Bronx, NY 10461, USA; ^4^ Longitudinal Study Section, Translational Gerontology Branch, Intramural Research Program, National Institute on Aging, National Institutes of Health, Bethesda, MD 20892, USA; ^5^ HudsonAlpha Institute for Biotechnology, Huntsville, AL 35806, USA; ^6^ Department of Medicine, Stanford University School of Medicine, Stanford, CA Stanford University School of Medicine, Stanford, CA 94305, USA; ^7^ Biostatistics, School of Public Health, University of California Los Angeles, Los Angeles, CA 90095, USA

**Keywords:** epigenetic clock, biological age, lung cancer

## Abstract

Lung cancer is considered an age-associated disease, whose progression is in part due to accumulation of genomic instability as well as age-related decline in system integrity and function. Thus even among individuals exposed to high levels of genotoxic carcinogens, such as those found in cigarette smoke, lung cancer susceptibility may vary as a function of individual differences in the rate of biological aging. We recently developed a highly accurate candidate biomarker of aging based on DNA methylation (DNAm) levels, which may prove useful in assessing risk of aging-related diseases, such as lung cancer. Using data on 2,029 females from the Women's Health Initiative, we examined whether baseline measures of “intrinsic epigenetic age acceleration” (IEAA) predicted subsequent lung cancer incidence. We observed 43 lung cancer cases over the nearly twenty years of follow-up. Results showed that standardized measures of IEAA were significantly associated with lung cancer incidence (HR: 1.50, *P* = 3.4×10^−3^). Furthermore, stratified Cox proportional hazard models suggested that the association may be even stronger among older individuals (70 years or above) or those who are current smokers. Overall, our results suggest that IEAA may be a useful biomarker for evaluating lung cancer susceptibility from a biological aging perspective.

## INTRODUCTION

Lung cancer is one of the most deadly of all cancers. While lung cancer accounts for only 14% of cancer incidence, it has an overall 5-year survival rate below 20% [1] and contributes to over a quarter of all cancer deaths [2]. In 2012 alone, an estimated 1.6 million people worldwide died from lung cancer. Lung cancer also carries a high financial burden, costing the United States about $12 billion, annually [3] and as a result, prevention of lung cancer has become a key area of focus in medical research. Decades of research has identified smoking as the leading preventable cause of lung cancer, for which it is estimated to contribute to nearly 90% of all cases [4]. Lung cancer was an exceedingly rare disease at the end of the 19^th^ century. However, the growing popularity of smoking during the 20^th^ century—particularly among males—gave way to rapidly increasing lung cancer rates. Tobacco smoke contains an array of chemicals, including a large number of genotoxic carcinogens, with the potential to cause mutations in essential genes, including those responsible for regulating cellular growth [5]. Not surprisingly, smoking history is the primary criterion used to decide who should undergo lung cancer screening with low-dose computed tomography (LDCT) [6].

In addition to smoking, chronological age is also a strong predictor of lung cancer risk. Like many other forms of cancer, lung cancer is considered an age-associated disease whose incidence rises steeply over the lifecourse, peaking around the seventh to eighth decade of life [1]. The link between lung cancer and age is hypothesized to arise in-part as a result of increasing accumulation of unrepaired damage [7] brought on by exposure to carcinogens, such as those found in cigarette smoke, as well as the age-related decline in immune system functioning [8] and increased cellular senescence [9]. Nevertheless, the rate of these changes significantly varies across individuals, and as a result, chronological age may not be the best proxy of the biological aging process underlying susceptibility to lung cancer incidence. DNA methylation levels at CpG dinucleotides may serve as a useful biomarker for assessing aging-related lung cancer susceptibility. Recently, we have developed a highly accurate candidate biomarker of aging based on DNA methylation (DNAm) levels [10], known as the “epigenetic clock”, which can be used to measure the age of human cells, tissues, and organs. Given that both smoking is seen as a pro-aging factor, and that lung cancer is an age-associated disease, a measure of epigenetic age acceleration could provide information about which individuals are at the highest risk of developing lung cancer.

Our previous work has shown that age acceleration effects are highly heritable [11], which suggests they could be capturing innate differences in the degree of energy allocation for maintenance and repair, which in turn influences the rate of physiological decline with age. Thus individuals with naturally decelerated aging rates may be less susceptible to exogenous toxins such as cigarette exposure. In this context, we examined whether intrinsic epigenetic age acceleration (IEAA)—which refers to epigenetic age acceleration adjusted for abundance measures of blood cell counts—predicts development of lung cancer. Different from typical epigenome wide association studies (EWAS), the current study involves a single hypothesis based on DNA methylation data: that a measure of epigenetic age acceleration predicts incidence of lung cancer. We hypothesize that variations in IEAA will account for differential risk of lung cancer, especially among current smokers and/or older adults (ages 70+), for whom lung cancer susceptibility is the greatest.

## RESULTS

### Sample characteristics

As shown in Table 1, the mean age of our samples at baseline was 65.3 years (s.d.=7.1). Standardized IEAA ranged from −4.3 to 8.5. Overall, approximately half of our sample was non-Hispanic white (47.7%), just under one-third (31.9%) were African American, and about 20% were Hispanic. The majority of our sample reported never smoking (54.4%), whereas 35.2% were former smokers, and 10.4% were current smokers. The mean number of pack-years for the full sample was 9.5 (s.d.=18.6), while the number of pack-years was 19.4 among former smokers and 25.9 among current smokers. Over the approximately 20 years of follow-up, we observed 28,688 total person-years and a total of 43 lung cancer incidences among the 2,029 participants in our sample.

**Table 1:** Sample Characteristics (*N* = 2,029)

Variable	Statistic
Standardized IEAA, Mean (S.D.)	0 (1)
Chronological Age, Mean (S.D.)	65.34 (7.10)
Non-Hispanic Black, Frequency (N)	0.32 (647)
Hispanic, Frequency (N)	0.20 (414)
Former Smoker, Frequency (N)	0.35 (714)
Current Smoker, Frequency (N)	0.10 (211)
Pack-Years Smoking, Mean (S.D.)	9.53 (18.55)
CHD Incidence, Frequency (N)	0.31 (646)
Lung Cancer Incidence, Frequency (N)	0.021 (43)
Person-Years, Total	28,688

### IEAA predicts lung cancer incidence

**Figure 1:**
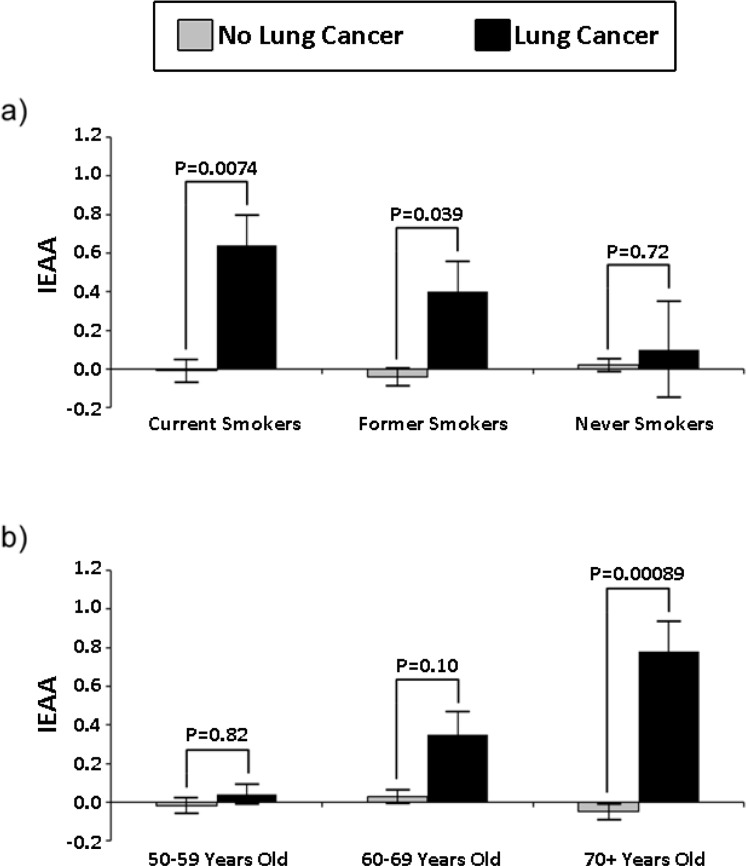
Smoking and age stratified barplots of standardized IEAA in cung cancer cases and controls.

The association between lung cancer and baseline IEAA was first examined using Kruskal Wallis tests. Results suggest that IEAA was significantly associated with subsequent lung cancer incidence for the full sample (*P* = 9.7×10^−4^). Additionally, in smoking and age stratified models (Figure 1), we found that IEAA was significantly associated with lung cancer incidence among current smokers (*P* = 7.4×10^−3^), former smokers (*P*=.039), and women in the oldest age group (70+) (*P* = 8.9×10^−4^). Results for current smokers and older women remain significant even after adjusting for multiple comparisons (Bonferroni *P* < 8.3×10^−3^).

Next we examined the associations using Cox proportional hazard models, adjusting for age, race/ethnicity, pack years, and smoking status. We found that IEAA at baseline significantly predicted lung cancer incidence (Table 2). The results using the full sample showed that a one unit increase in IEAA was associated with a 50% increase in the risk of developing lung cancer (HR: 1.50; *P* = 3.4×10^−3^). Results for age-stratified models also showed that IEAA was more predictive of lung cancer incidence in older compared to younger age groups. For instance, among participants who were ages 50–59 at baseline, there was no association between IEAA and lung cancer incidence (HR: 0.94; *P* = 0.91). However among those 60–69 years of age at baseline there was a marginal association (HR: 1.35; *P* = 0.11) found, and among those 70–79 years old at baseline there was a statistically significant association between baseline IEAA and subsequent lung cancer incidence (HR: 2.51; *P* = 7.7×10^−4^), such that a one unit increase in IEAA was associated with an over two and a half fold increase in the risk of developing lung cancer.

**Table 2:** Cox proportional hazard model of lung cancer, by age

	Hazard Ratio (P-Value)			
	All Ages	50–59	60–69	70+
IEAA	1.50 (3.4×10^−3^)	0.94 (0.91)	1.35 (0.11)	2.51 (7.7×10^−4^)
Age	1.09 (2.0×10^−3^)	1.45 (0.17)	1.11 (0.19)	1.26 (0.05)
Black	0.87 (0.73)	1.12 (0.94)	0.64 (0.37)	1.88 (0.46)
Hispanic	1.25 (0.67)	0.90 (0.95)	0.72 (0.68)	6.53 (0.04)
CHD	0.64 (0.22)	0.00 (0.99)	0.61 (0.27)	0.90 (0.87)
Former Smoker	2.22 (0.09)	2.39 (0.53)	2.35 (0.18)	2.02 (0.41)
Current Smoker	6.17 (3.8×10^−4^)	3.22 (0.44)	5.38 (0.02)	14.78 (3.2×10^−3^)
Pack Years	1.03 (1.7×10^−7^)	1.01 (0.56)	1.03 (1.1×10^−4^)	1.04 (2.1×10^−3^)
N	2,029	505	947	577
Events	43	4	27	12
Total Person-Years	28,688	7,595	13,540	7,554
R^2^	0.036	0.012	0.045	0.054

**Table 3:** Cox proportional hazard model of lung cancer, by smoking status

	Hazard Ratio (P-Value)		
	Current Smokers	Former Smokers	Never Smokers
IEAA	2.06 (6.1×10^−3^)	1.41 (0.11)	1.21 (0.60)
Age	1.15 (0.02)	1.04 (0.31)	1.14 (0.03)
Black	0.69 (0.65)	0.86 (0.77)	1.17 (0.90)
Hispanic	2.06 (0.65)	0.00 (0.99)	6.79 (0.02)
CHD	0.53 (0.33)	0.38 (0.12)	1.52 (0.57)
Pack Years	1.04 (2.3×10^−3^)	1.02 (2.4×10^−4^)	
N	211	714	1,104
Events	13	22	8
Total Person-Years	2,799	10,015	15,875
R2	0.122	0.037	0.008

**Figure 2:**
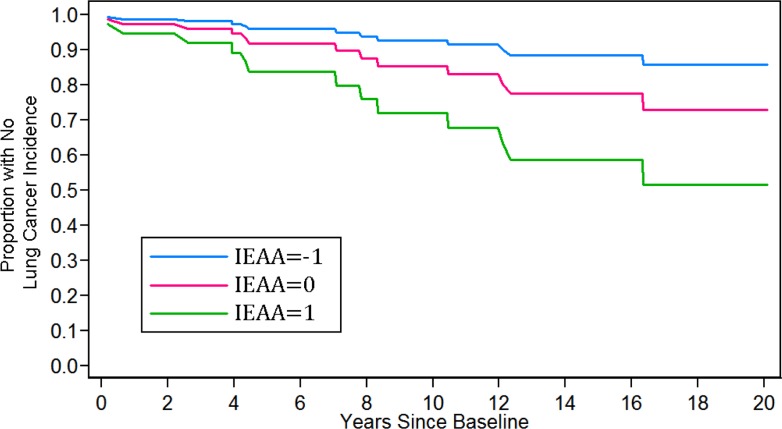
Kaplan-Meier curves for 20-year lung cancer incidence.

**Figure 3:**
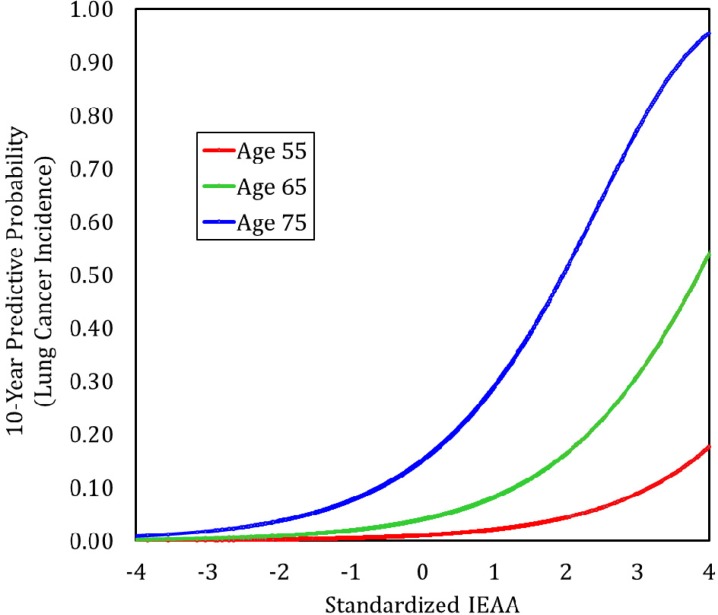
Predicted 10-Year lung cancer incidence by age and IEAA

We used the results from our Cox models to calculate Kaplan-Meier curves (lung cancer incidence) for women ages 70–79. These curves were calculated assuming 1) a chronological age of 75, 2) Non-Hispanic white race/ethnicity, 3) current smoking status, and 4) having 30 pack-years of smoking history. Three curves were calculated, varying the level of baseline IEAA (standardized) so that it equaled −1, 0, and 1, respectively. As shown in Figure 2, having a standardized IEAA level equal to one greatly increased the likelihood of developing lung cancer over twenty years of follow-up. For instance, after ten years, only about 5% of individuals in the negative age acceleration group (IEAA=−1) were predicted to develop lung cancer, and after twenty-years the number was only predicted to rise to about 10%. In the average age acceleration group (IEA*A* = 0), about 12%, and 25% of individuals were predicted to develop lung cancer after ten and twenty years, respectively. However, for women with positive age acceleration (IEA*A* = 1), it was predicted that after ten years almost 25% would develop lung cancer, and after twenty years, over half would have developed lung cancer. When examining these trends in younger groups as well, not only was lung cancer risk lower overall, but IEAA did not have as strong an effect on lung cancer incidence (Figure 3). For instance, estimates suggest that among those with negative age acceleration, the 10-year lung cancer incidence would only be 0.5% for smokers age 55, and 2% for smokers age 65. If they had average age acceleration, the 10-year lung cancer incidence was predicted to be only 1% for smokers age 55, and 4% for smokers age 65. Finally, for those with positive age acceleration, the 10-year lung cancer incidence was estimated to be 2% for smokers age 55 and 8% for smokers age 65.

Next we examined the association between IEAA and lung cancer incidence, stratified by smoking status, and adjusting for chronological age, race/ethnicity, CHD, and pack-years (except in the model for never smokers). We found that overall, IEAA was most predictive in current smokers, which also (as expected) was the group with the highest incidence rate of lung cancer (5.9%). Our results showed that for current smokers, a one unit increase in standardized IEAA was associated with an over two-fold increase in the risk of developing lung cancer (HR: 2.06; *P* = 6.1×10^−3^). For former smokers, who had a lung cancer incidence rate of about 3%, epigenetic age only marginally predicted lung incidence (HR: 1.40; *P* = 0.11), and finally for never smokers, who had an incidence rate of only 0.7%, there was no association found (HR: 1.21; *P* = 0.60) between IEAA and lung cancer risk.

### IEAA & smoking history

**Figure 4:**
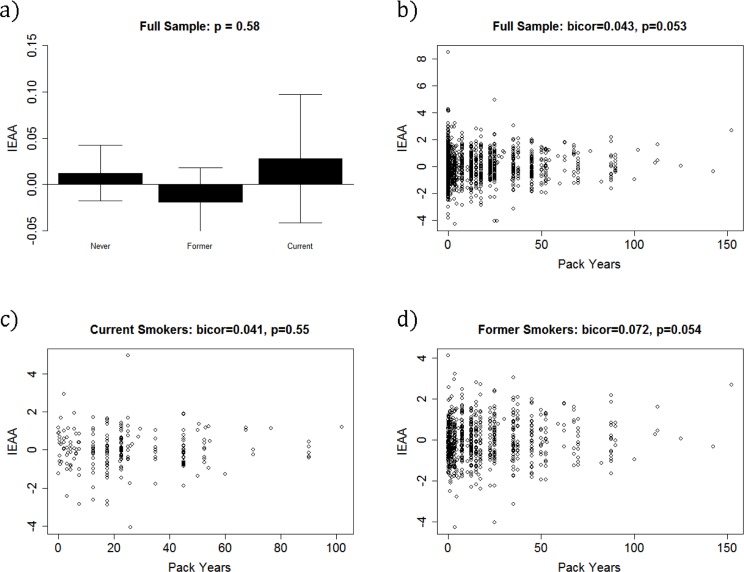
Baseline IEAA by smoking status and pack-years.

Finally, to assess whether cigarette use increased IEAA, we used the Kruskal Wallis test and biweight midcorrelation to examine the association between IEAA and both smoking status and pack-years. Barplots (Figure 4) showed no association between IEAA and smoking status (*p* = 0.58). Using biweight midcorrelation we also examined whether higher pack-years of smoking was associated with an increase in IEAA. Overall, we found a positive but weak association when considering all participants (bico*r* = 0.043, *p* = 0.053) and former smokers (bico*r* = 0.072, *p* = 0.054) but no association among current smokers (bico*r* = 0.041, *p* = 0.55).

## DISCUSSION

We have shown that a blood based measure of accelerated aging (IEAA) is a significant prognosticator of lung cancer incidence in a multi-ethnic sample of women. This suggests that IEAA may serve as a useful marker of the aging-related decline that influences lung cancer susceptibility, particularly among individuals exposed to high levels of cigarette smoke. Our results indicated that having an aging acceleration rate that is one standard deviation above the mean (standardized IEA*A* = 1) is associated with as a high as a 2.5-fold increase in the risk of developing lung cancer. Given the lethality of lung cancer, identifying susceptible individuals early is essential.

Biomarkers which capture biological signals representing susceptibility could aid both primary and secondary prevention strategies for lung cancer by 1) raising awareness and influencing positive behavioral change among high-risk individuals, and 2) facilitating targeted screening and prevention strategies [12]. LDCT imaging aimed at early detection of lung cancer is a promising prevention strategy; however, screening criteria remains solely based on demographic and behavioral characteristics, particularly smoking history and age [6]. The use of high-dimensional omics markers has the potential to inform eligibility criteria and facilitate prevention. For instance, while we know that smoking status is the largest predictor of lung cancer, and that the likelihood of incidence rises steeply with age, significant variation after accounting for age and smoking still exist. These differences may stem from individual-level variations in the rate of physiological alterations and system dysregulation that precede cancer pathogenesis, and ultimately could reflect differences in the rate of biological aging.

Aging is thought of as a time-dependent decline in system functioning, putting the organism at increased risk of death and disease [13]. Thus, under specific environmental circumstances, aging could enable the development of diseases such as lung cancer. There is evidence suggesting aging and cancer are inherently linked [14]. Cancer incidence is strongly age-dependent—the majority of lung cancer incidences occur among individuals who are 65 years or older [15]. There is speculation that both cancer and aging may occur as a result of damage accumulation and genomic instability, which if unresolved, can cause physiological degradation and contribute to cancer cell formation [7]. Additionally, interventions such as caloric restriction, which have been shown to retard aging in model organisms, also appear to have strong effects on cancer incidence, progression, and metastasis [16]. Together this could be taken to suggest individuals who age at an accelerated rate, have increased risk of cancer, including lung cancer, compared to their slower aging peers.

The link between aging and cancer is consistent with our results showing that participants who are epigenetically older have a higher incidence of lung cancer. Our model predicted that only 5% of older smokers with negative age acceleration (standardized IEAA equal to −1) would develop lung cancer over the following ten years, compared to 12% of older smokers with average age acceleration (IEA*A* = 0), and 25% of older smokers with positive age acceleration (standardized IEAA equal to 1). We also showed that the ability for IEAA to predict lung cancer was strongest among individuals ages 70 and older. A one unit difference in IEAA was associated with a 2.5-fold increase in lung cancer among individuals ages 70+, compared to an only 50% increase when considering the entire 50+ year old sample. The ability of IEAA to predict lung cancer among the oldest age group is most likely due to the fact that this is the age group for whom lung cancer risk is the greatest. In the U.S., incidence of lung cancer has been shown to peak around ages 75 to 79—with 80% of lung cancer mortality occurring in individuals ages 65 and older, and 20% occurring in those who are 80 years or older [15]. Our results also show that lung cancer is most common in the oldest age groups and that this is particularly true among those with accelerated aging. For instance, among individuals who were epigenetically younger than expected (IEAA=−1) it was predicted that 0.5% of smokers age 55, 2% of smokers age 65, and 7.5% of smokers age 75 would develop lung cancer over the next ten years. However, among individuals who were epigenetically older than expected (IEA*A* = 1) it was predicted that 2% of smokers age 55, 8% of smokers age 65, and 29% of smokers age 75 would develop lung cancer over the next ten years. This likely ties back to the aging-dependent nature of lung cancer. Middle-aged individuals with accelerated aging phenotypes, may not have reached the point where they are biologically old enough to increase their risk of lung cancer incidence. On the other hand, older individuals with accelerated epigenetic aging, may have crossed the threshold that, under particular environmental circumstances such as smoking, puts them at risk of developing lung cancer. *In vivo* studies in mice showed that there was no effect of Bin1 ablation on cancer incidence in mice who were less than or equal to 12 months of age; however lung adenocarcinomas were present in half of the mosaic mice who were 18 to 20 months of age [17]. Additionally, an *in vivo* study using rats showed that intravenously administered rhabdomyosarcoma cells had increased colony forming capacity in lung if administered to old (15-month) rather than middle-aged (12-month) animals [18]. Together, these findings suggest that lung cancer incidence may results from exposure to endogenous carcinogens in the presence of aging-associated epigenetic alterations. This potential assumption is further supported by our findings from smoking-stratified models where we found a strong significant association between IEAA and lung cancer among smokers, compared to an only moderate association for former smokers and no association for never smokers. Furthermore, smoking status and pack-years was not associated with IEAA. This suggests that IEAA does not mediate the association between smoking and lung cancer, but rather that IEAA may only influence lung cancer if a person is a smoker. This could also mean that smoking is a bigger risk-factor for people who have accelerated aging phenotypes. If validated, this could be a useful marker for targeting smoking cessation interventions. Given that epigenetic age acceleration has been shown to be highly heritable [11], these findings could signify innate differences in susceptibility to endogenous stressors. Large-scale multinational genome-wide association studies (GWAS) of the genetic variation associated with lung cancer initially found that the 5p15 and 15q25 regions were associated with risk of lung cancer among smokers [19-24]. In moving forward, it may be useful to determine if genetic loci which influence lung cancer risk and longevity in smokers are associated with differences in IEAA.

Our study has several limitations which need to be acknowledged. First, our small number of lung cancer cases (*n* = 43) prevented us from further stratifying our models (e.g. looking at the effect of IEAA in 70+ year old current smokers). Thus, validation of our findings in independent samples remains an important next step for understanding the relationship between IEAA and lung cancer. Second, our study was restricted to females who have lower rates of smoking and lung cancer compared to males in the U.S. Nevertheless, exposure to second-hand smoke could contribute to higher rates of lung cancer among non-smokers in our female-only sample, compared to a male-only sample. Third, we did not have data on histological subtype, or stage of lung cancer at diagnosis. Fourth, due to the sampling procedures, our-time to event analysis could be biased given that inclusion in the sample is dependent upon survival to 2010. Nevertheless, we also conducted analysis using logistic regression models and ran Cox models excluding cases diagnosed within 1, 3, and 5 years of baseline. Both procedures produced analogous results to what was reported.

The epigenetic clock has been shown to predict other aging-related outcomes, such as all-cause mortality [25], and cognitive and physical functions [26]. Further, it was used to show that 1) Down syndrome is associated with accelerated aging effects [27], 2) the cerebellum ages slowly [28], and 3) that the blood of subjects with a severe developmental disorder ages normally [29]. However, to the best of our knowledge, our study is the first to show its ability to predict future onset of lung cancer. Given that lung cancer has the highest cancer mortality rate, identifying susceptibility markers has the potential to extend life expectancy and improve quality of life through early detection and diagnosis. Our study demonstrates that a surrogate tissue (blood) lends itself for detecting accelerated aging effects that predispose the malignant transformation of other tissues (such as lung). Currently, we don't have any evidence that epigenetic aging effects in blood tissue lend themselves for prognostication of other kinds of cancers. If IEAA is found to be causal, rather than a byproduct of another causal pathway in lung cancer, alteration in CpG methylation could prove to be an effective method for preventing lung cancer among at-risk populations. Further, investigating this association in other populations (especially males) to establish whether lung cancer susceptibility loci operate through IEAA, and to determine whether interventions that modify methylation can decrease lung cancer risk, represent important next steps.

## METHODS

### Sample description

Participants included a subsample of 2,029 participants of the Women's Health Initiative (WHI) study, a national study that began in 1993, which enrolled postmenopausal women between the ages of 50–79 years [30]. Women who were ineligible to participate in the trials or who chose not to be randomized were invited to participate in the observation arm of the study. Participants selected for this study were part of an integrative genomics study with a primary aim of identifying novel genomic determinants of CHD. Thus, a case-control sampling design was adopted. All cases and controls were required to have already undergone genome wide genotyping at baseline as well as profiling of seven cardiovascular biomarkers as dictated by the aims of other ancillary WHI studies. The study design also resulted in oversampling of African American and Hispanics.

### Smoking status

Smoking history was assessed at baseline from self-reports. Participants were first asked whether or not they had smoked at least 100 cigarettes in their lifetime. Those reporting ‘no’ to this question were classified as never smokers. Women reporting that they had smoked at least 100 cigarettes in their lifetime were then asked whether they smoked cigarettes now. Those who answered ‘yes’ were classified as current smokers and those who answered ‘no’ were classified as former smokers. Additionally, our study also utilized a variable for pack-years of smoking, which was based on current and former smokers' self-reports of the number of years they actively smoked, as well as the average number of cigarettes smoked per day.

### Lung cancer incidence

Incident lung cancer cases were defined as either the first occurrence of lung cancer or a death due to lung cancer. Incidences were self-reported during annual health updates. Additionally, medical records were used to verify lung cancer incidence after being reviewed by physician adjudicators [31]. Characteristics of lung cancer were coded in accordance with the International Classification of Diseases for Oncology (ICD-O_2) from the Surveillance Epidemiology and End Result (SEER) [32]. National Death Index searches were performed to improve mortality ascertainment and lung cancer mortality was also verified via review of death certificates. Person-days for lung cancer incidence—which we converted to person-years, using precision to four decimal places—was also recorded by the WHI. Finally, participants with no reported lung cancer incidence or death were censored and the number of days (converted to years) between baseline and their last day of observation in the WHI was set as their observation time.

### DNA methylation data from blood

Methylation analysis was performed at HudsonAlpha Institute of Biotechnology using the Illumina Infinium Human-Methylation450 BeadChip. The Illumina BeadChips measures bisulfite-conversion-based, single-CpG resolution DNA methylation levels at 485577 different CpG sites in the human genome. These data were generated by following the standard protocol of Illumina methylation assays, which quantifies methylation levels by the β value using the ratio of intensities between methylated and un-methylated alleles. Specifically, the β value is calculated from the intensity of the methylated (M corresponding to signal A) and un-methylated (U corresponding to signal B) alleles, as the ratio of fluorescent signals β = Max(M,0) / [Max(M,0) + Max(U,0) + 100]. Thus, β values range from 0 (completely un-methylated) to 1 (completely methylated) (Dunning, 2008).

### Intrinsic Epigenetic Age Acceleration (IEAA) in blood

We used the 353 CpGs and coefficient values reported in [11] to define DNAm age. These CpGs and coefficient values were chosen in independent data sets by regressing age on CpGs using the elastic net penalized regression model (implemented in the R package glmnet) [33]. DNAm age is defined as predicted age, in years.

Based on DNAm age, one can define several epigenetic measures of age acceleration, e.g. one can regress DNAm age on chronological age and form residuals. However, the resulting measure may still show some relationship to blood cell counts. Instead, we focus here on a measure of intrinsic epigenetic age acceleration (IEAA) where the term “intrinsic” implies that it is unconfounded by differences in blood cell types. Measures of IEAA are attractive for this study since they measure pure, unconfounded epigenetic aging effects, rather than measuring the age-related functional decline of the immune system—in blood cell composition such as the decrease of naive CD8+ T cells and the increase in memory or exhausted CD8+ T cells [34-37]. This measure, which is also known as "age acceleration adjusted for blood cell counts" and denoted by AAHOAdjCellCounts in our software, is defined as residual resulting from regressing DNAm age on chronological age and seven measures of blood cells counts including: naive CD8 T cells, exhausted CD8 T cells, plasma B cells (effector B cells), CD4 T cells, natural killer cells, monocytes, and granulocytes. The abundance measures of blood cells were imputed based on DNA methylation data as described in [38]. For IEAA, a positive value indicates that DNA methylation age is higher than expected given the individual's chronological age (accelerated aging), whereas a negative value indicates that DNA methylation age is lower than expected given the individual's chronological age (decelerated aging).

### Statistical analysis

Kruskal Wallis tests were used to initially examine the association between IEAA at baseline and subsequent lung cancer incidence. Next, Cox proportional hazard models were used to test whether differences in IEAA predicted incidence of lung cancer. These models were run on the full sample of participants—adjusting for age, race/ethnicity, CHD status, pack-years and smoking status (never, former, current)—and stratifying by 1) 10-year age groups (50–59, 60–69, 70–79) and 2) smoking status. Based on these models, we calculated predictive probabilities for 10-year incidence of lung cancer based on IEAA. We adjusted for CHD status, given that it was an important inclusion criteria when the pilot sample was selected.
